# Probing the Spatial Homogeneity of Exfoliated HfTe_5_ Films

**DOI:** 10.1021/acsnano.4c02081

**Published:** 2024-07-03

**Authors:** Maanwinder P. Singh, Qingxin Dong, Gen-Fu Chen, Alexander W. Holleitner, Christoph Kastl

**Affiliations:** † Walter Schottky Institute and Physics Department, Technical University of Munich, Am Coulombwall 4a, 85748 Garching, Germany; ‡ Munich Center for Quantum Science and Technology (MCQST), Schellingstr. 4, 80799 Munich, Germany; § Institute of Physics and Beijing National Laboratory for Condensed Matter Physics, Chinese Academy of Sciences, 100190 Beijing, China; ∥ School of Physical Sciences, University of Chinese Academy of Sciences, 100049 Beijing, China; ⊥ Songshan Lake Materials Laboratory, Dongguan 523808, Guangdong, China

**Keywords:** van der Waals materials, Raman microscopy, strain, topological insulator, disorder

## Abstract

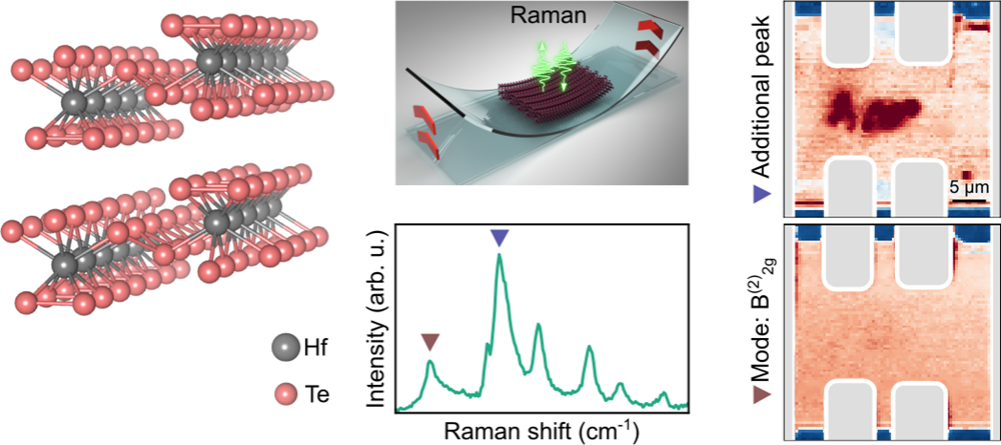

In van der Waals materials, external strain is an effective tool to manipulate and control electronic responses by changing the electronic bands upon lattice deformation. In particular, the band gap of the layered transition metal pentatelluride HfTe_5_ is sufficiently small to be inverted by subtle changes of the lattice parameters resulting in a strain-tunable topological phase transition. In that case, knowledge about the spatial homogeneity of electronic properties becomes crucial, especially for the microfabricated thin film circuits used in typical transport measurements. Here, we reveal the homogeneity of exfoliated HfTe_5_ thin films by spatially resolved Raman microscopy. Comparing the Raman spectra under applied external strain to unstrained bulk references, we pinpoint local variations of Raman signatures to inhomogeneous strain profiles in the sample. Importantly, our results demonstrate that microfabricated contacts can act as sources of significant inhomogeneities. To mitigate the impact of unintentional strain and its corresponding modifications of the electronic structure, careful Raman microscopy constitutes a valuable tool for quantifying the homogeneity of HfTe_5_ films and circuits fabricated thereof.

Topology provides a framework for classifying phases of matter based on their quantum geometrical band properties beyond the established concepts of symmetry breaking or order parameters.^1−4^ For example, in insulators, the transition from the trivial to the topological phase arises solely from the inversion of the band gap at high symmetry points in the Brillouin zone. Band inversions can be driven by spin–orbit interactions,^5^ quantum confinement effects,^6,7^ or strain,^8^ with corresponding band gaps from hundreds down to tens of meV. Particularly for weakly gapped or semimetallic systems, applied strain induces topological band inversions already by comparatively small shifts of band energies.^8^ In this context, the transition metal pentatelluride HfTe_5_, and similarly ZrTe_5_, is an interesting material with a low-energy bulk dispersion described by weakly gapped 3D Dirac Fermions at the Γ-point with gap sizes on the order of 5–10 meV,^9−14^ with low carrier densities down to 8 × 10^15^ cm^–3^, and with low-temperature mobilities reaching 1 × 10^6^ cm^2^V^–1^s^–1^.^10,11,15^ Notably, their electronic structure resides at the transition between a strong and weak topological insulator.^9,16−18^ The topological character of the gap has been shown to be strain-tunable with evidence from band structure calculations as well as transport and spectroscopic experiments.^19−21^ While these studies demonstrate the general possibility to dynamically and reversibly control transitions between topological phases via strain in HfTe_5_ and ZrTe_5_, the important aspect of sample homogeneity in micro- to nanoscale devices has not been elucidated so far.

Here, we demonstrate that the homogeneity of exfoliated and contacted HfTe_5_ films varies considerably on a micrometer scale using confocal Raman microscopy. To this end, we locally image strain-related variations of the Raman modes with a submicron spatial resolution. Comparing the Raman spectra of exfoliated HfTe_5_ films under an applied external strain to reference measurements on unstrained HfTe_5_ bulk crystals, we are able to correlate the local variations of Raman signatures to inhomogeneous strain profiles in the sample imparted during the exfoliation as well as the contact fabrication process. On the one hand, our results imply that inhomogeneities within the films have important implications for the interpretation of sometimes seemingly disparate results on microscale contacted films. On the other hand, we argue that Raman microscopy can be a valuable tool to pre- or postselect films with optimized homogeneity for further advanced experiments, e.g., on the topological properties of the materials and their circuits.^20,21^

## Results

**Figure 1:**
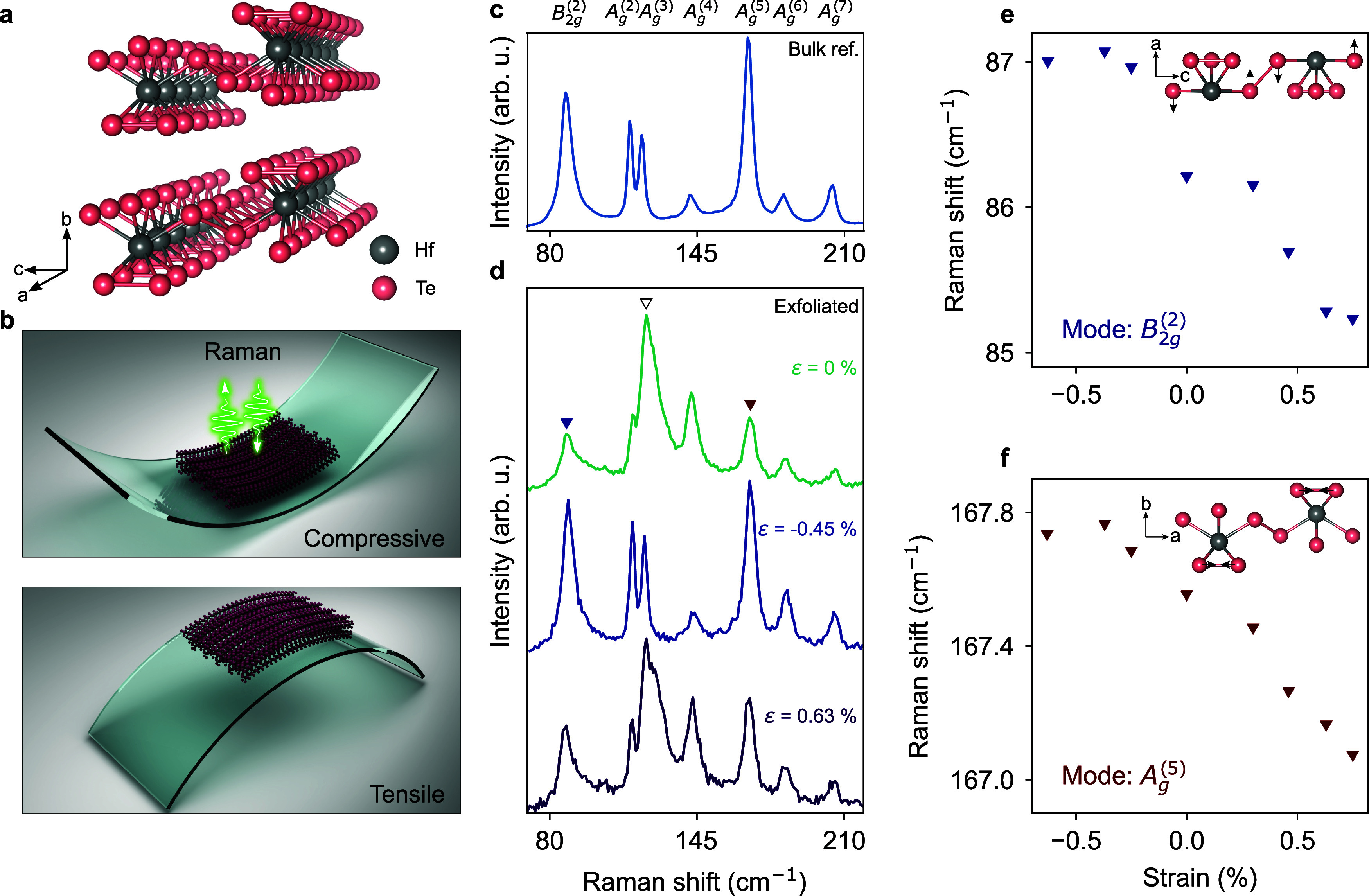
Impact of uniaxial strain on the Raman spectrum of exfoliated HfTe_5_ films. (a) Crystal structure of HfTe_5_. (b) Schematics of a strain device based on a flexible substrate. The crystals are oriented such that compressive (top) and tensile (bottom) strain is applied along the *a*-direction. (c) Raman spectrum of an unstrained bulk reference crystal. (d) Raman spectra of an exfoliated HfTe_5_ film (thickness = 1.48 μm) under three distinct conditions: nominally unstrained (ϵ = 0%), compressive strain (ϵ = −0.45%), and tensile strain (ϵ = 0.63%). Peak center of (e) B_2*g*_^(2)^ and (f) A_*g*_^(5)^ modes under strain. The insets depict the dominant atomic displacements of the corresponding phonons. All measurements at room temperature.

Starting point are Raman measurements on exfoliated films (thickness about 1 μm, lateral dimensions about 10 μm) with an applied external strain, which we compare to reference measurements on bulk, as-grown crystals (thickness larger than 100 μm, lateral dimensions about 1 mm). HfTe_5_ has an orthorhombic crystal structure (Figure 1a) with symmetry space group C_mcm_ (D_2*m*_^17^).^22^ The material consists of quasi-one-dimensional, covalently bonded chains in the *a*-direction, which are linked by comparatively weak bonds along the *c*-direction. The van der Waals layers are then stacked along the *b*-axis. Consequently, exfoliated crystals are typically rectangular with the long and short axes aligned along the *a*- and *c*-direction, respectively (Figure 1b). The crystal’s primitive unit cell contains 12 atoms, which leads to 36 normal modes at the center Γ of the Brillouin zone.^23,24^ Only 18 modes are Raman active, which are 6A_*g*_ + 4B_1*g*_ + 2B_2*g*_ + 6B_3*g*_ modes. With the light incident along the *b*-direction, perpendicular to the layered film, six A_*g*_ and two B_2*g*_ modes are experimentally accessible.^23,24^ Figure 1c shows the Raman spectrum of a bulk reference crystal grown by chemical vapor transport.^25^ Out of the seven observed modes, six align with the established literature, comprising five A_*g*_ modes and one B_2*g*_ mode. An additional mode occurs at 206 cm^–1^. Although this particular mode does not exactly match theoretical expectations, it was tentatively assigned as the A_1_ diatomic mode in the previous literature.^24^ To apply uniaxial strain along the *a*-axis of the exfoliated HfTe_5_ thin films, we use a standard two-point bending geometry with a flexible polymer substrate (Figure 1b and Methods).

Figure 1d presents a comparison of the Raman spectrum for a HfTe_5_ film, which was exfoliated from the bulk reference crystal, under conditions of unstrained, compressive, and tensile strain, respectively. Already in the nominally unstrained configuration (ϵ = 0%), the Raman spectrum of the exfoliated crystal differs notably from the bulk Raman spectrum. The A_*g*_^(2)^ and A_*g*_^(3)^ modes can barely be separated, and a large additional shoulder appears in the spectrum (open triangle in Figure 1d). However, under an applied compressive strain (ϵ = −0.45%), the A_*g*_^(2)^ and A_*g*_^(3)^ modes become distinguishable, the additional shoulder vanishes and the overall spectrum resembles precisely the bulk reference. Upon reversing the strain from compressive to tensile (ϵ = +0.63%), the A_*g*_^(2)^ and A_*g*_^(3)^ modes become indistinguishable again, and the additional shoulder reappears.

To quantify the impact of the applied uniaxial strain in more detail, we analyze the Raman shifts for the two most prominent modes, namely, B_2*g*_^(2)^ and A_*g*_^(5)^ (highlighted by the dark blue and dark red triangles in Figure 1d). Both Raman modes blueshift under compressive and redshift under tensile strain in a linear fashion (Figure 1e,f), as is expected for stiffening (softening) of the lattice under compression (tension). Experimentally, we find that the B_2*g*_^(2)^ mode exhibits a significantly larger shift with strain compared to the A_*g*_^(5)^ mode (on the order of 2% compared to 0.04%). This can be understood intuitively by considering the bonds and atoms participating in the phonon motion. For the B_2*g*_^(2)^ mode, the oscillating tellurium atoms interlink the quasi-one-dimensional chains along the *a*-axis. Therefore, the oscillation is very sensitive to changes of the lattice along the *a*-axis (inset of Figure 1e). By contrast, for the A_*g*_^(5)^ mode, the oscillating tellurium atoms are coordinated mainly to the hafnium atom resulting in a comparatively weaker sensitivity to strain along the *a*-axis (inset of Figure 1f).

**Figure 2:**
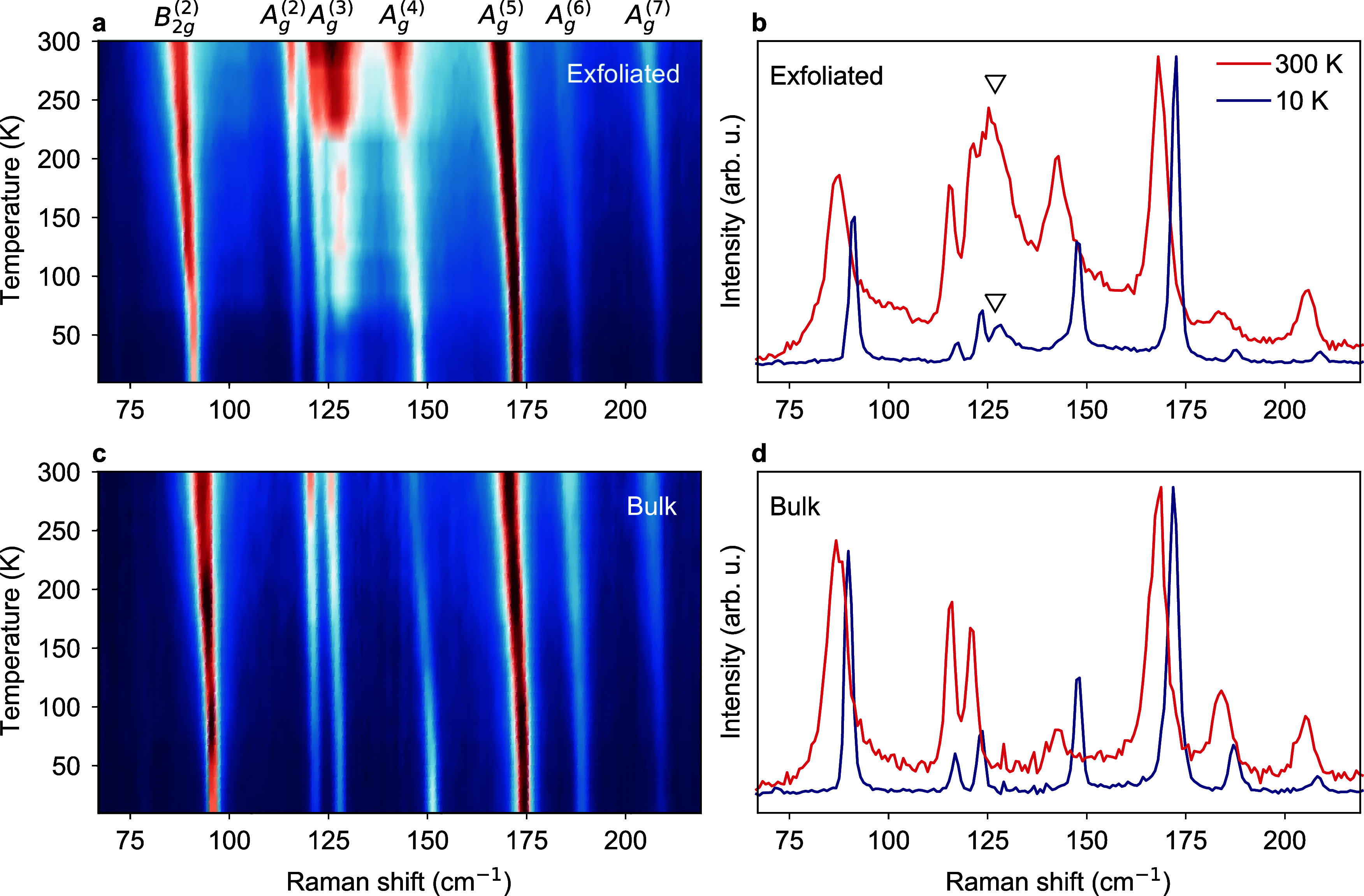
Temperature-dependent Raman spectra of HfTe_5_. Raman spectra for (a, b) exfoliated film (thickness = 1.3 μm) and (c, d) bulk reference crystal (thickness > 100 μm) from 300 to 10 K. The Raman spectrum of the exfoliated film exhibits an additional peak next (open triangle) to the A_*g*_^(3)^ mode, which is not present in the bulk reference spectrum. All spectra are normalized to the amplitude of the A_*g*_^(5)^ mode.

Having established the impact of externally applied uniaxial strain, we turn our focus to the impact of temperature-induced changes of the lattice parameters on the Raman modes. We note that special care has to be taken in choosing an appropriate laser power, in particular for confocal microscopy with small laser spot sizes and high power densities. By comparing the temperature dependence to the laser power dependence, we find that under 1 mW excitation, which is a common value from literature,^26,27^ the local temperature within the sample volume probed by the laser spot can be up to 100 K above the substrate temperature (Figure S1). In turn, the determined positions of the Raman peaks can deviate by several wavenumbers from their true value because of the large discrepancy between the substrate and crystal temperature. For all our measurements, we took care to minimize the local heating by using a laser power *P*_laser_ ≤ 100 μW. Under these conditions, we find that the uncertainty on the true peak position due to local laser heating, in addition to the statistical fitting error, is about 0.1 cm^–1^. Figure 2 depicts the temperature dependence of the Raman spectrum from 300 K down to 10 K for a HfTe_5_ film exfoliated on a Si/SiO_2_ substrate (Figure 2a,b) versus the bulk reference crystal on a Si/SiO_2_ substrate (Figure 2c,d). All Raman spectra are normalized to the amplitude of the A_*g*_^(5)^ mode for better comparison of relative intensity changes. Similar to Figure 1, the spectrum of the exfoliated film exhibits an additional peak (open triangle in Figure 2b) adjacent to A_*g*_^(3)^ at room temperature. Upon cooling the substrate to 10 K, the amplitude of this peak decreases significantly, such that at the lowest temperature both A_*g*_^(2)^ and A_*g*_^(3)^ are well resolved (Figure 2b). The observed temperature dependence is consistent with our strain measurements. In both cases, a stiffening of the lattice, either due to the thermal contraction or external compressive strain, suppresses the additional peak. The bulk reference spectrum remains qualitatively unchanged as the temperature decreases except for the anticipated narrowing and blueshift of all Raman modes (Figure 2c,d).

**Figure 3:**
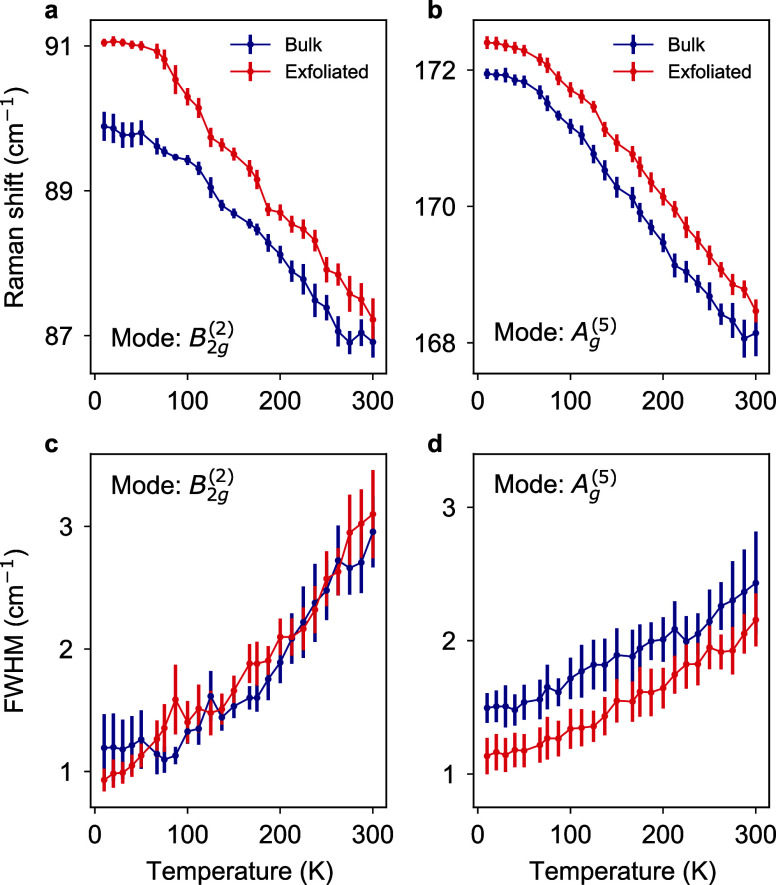
Temperature-dependent shift and narrowing of Raman modes. (a, b) Position and (c, d) full width at half-maximum (fwhm) of the B_2*g*_^(2)^ and A_*g*_^(5)^ Raman modes in the bulk and exfoliated HfTe_5_ crystal. The parameters are determined from a standard peak fitting procedure, and the error bars denote the estimated 3σ confidence intervals.

At first sight, the strain and temperature dependence suggests a built-in tensile strain in the exfoliated samples, which manifests as the additional peak next to the A_*g*_^(3)^ mode. Consistently, compression of the lattice always suppresses this peak. However, closer inspection of the absolute energies of the Raman modes reveals that the spectrum of this specific exfoliated sample is blue-shifted with respect to the bulk reference (Figure 2b,d). The blueshift becomes immediately evident in Figure 3a,b, where we show the temperature evolution of the B_2*g*_^(2)^ and A_*g*_^(5)^ modes as determined by a standard peak fitting procedure. The modes of the exfoliated film exhibit a consistent blueshift across the full temperature range, which must be interpreted as an overall compressive strain of the exfoliated film relative to the bulk reference (cf. Figure 1e,f). To rule out differences in sample quality, we compare the fitted widths of the B_2*g*_^(2)^ and A_*g*_^(5)^ modes between the exfoliated film and the bulk reference (Figure 3c,d). The peaks of the exfoliated sample are equally narrow or even narrower indicating a comparable or even improved crystal quality.

**Figure 4:**
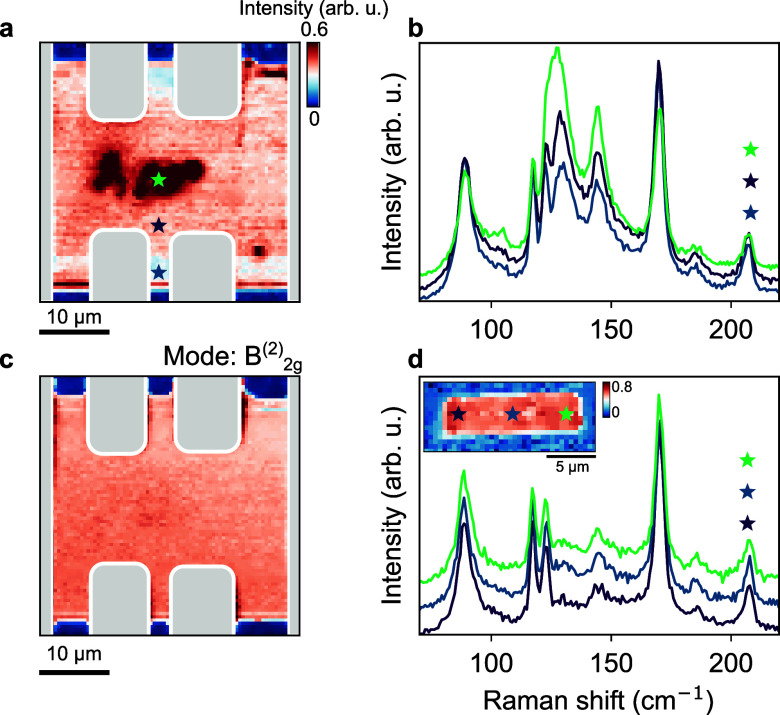
Mapping the spatial homogeneity of exfoliated HfTe_5_ crystals. (a) Raman intensity map of a contacted sample at 300 K. The intensity was integrated from 118 to 138 cm^–1^. The gray-shaded areas denote gold contacts. The mapping reveals localized hotspots of enhanced Raman intensity corresponding to inhomogeneous strain patterns, potentially originating from the contacts. (b) Raman spectra corresponding to the positions highlighted by star symbols in (a). The spectra are offset vertically for clarity. (c) Raman intensity map of a contacted sample at 300 K. The intensity was integrated from 60 to 110 cm^–1^ corresponding to the intensity of the B_2*g*_^(2)^ mode. Within this spectral range, the sample area appears homogeneous. (d) Inset shows a Raman intensity map of an uncontacted crystal (sample thickness = 120 nm) located on the very same substrate as the crystal in (a). The Raman spectra correspond to the positions highlighted by the star symbols. Compared to the contacted film, the noncontacted film is spatially more uniform and homogeneous, suggesting a detrimental impact of the contact formation process on the sample homogeneity.

To reconcile the strain data, which at first sight suggests the additional peak to be a signature of built-in, uniaxial tensile strain, with the temperature dependence, which demonstrates the coexistence of the additional peak and overall, built-in compressive strain, we consider the homogeneity of the exfoliated films. Figure 4a shows a Raman intensity map of the exfoliated crystal at 300 K. Note that this sample is the same as discussed in Figures 2 and 3. To obtain the intensity map, the spectrum was integrated between 118 and 138 cm^–1^ corresponding to the region where we observe the additional peak in the spectrum. Strikingly, the Raman intensity reveals a strongly inhomogeneous distribution, with distinct hot spots close to the center of the crystal. The gray-shaded regions mark gold contacts, which were evaporated after the exfoliation process (see Methods and ref (28)). Comparison of the full Raman spectra at three distinct positions (marked by the stars in Figure 4a) reveals indeed that the hot spots coincide with an enhanced intensity of the additional peak (Figure 4b, the spectra are offset vertically for clarity). By contrast, when imaging the intensity of the B_2*g*_^(2)^ mode, the sample appears spatially homogeneous (Figure 4c). The B_2*g*_^(2)^ mode was chosen for comparison because it exhibits a similar trend in temperature (Figure S2). To rule out bubbles or wrinkles as the source of the hotspots, we mapped out the topography of the very same exfoliated crystal using atomic force microscopy (Figure S3). We do not observe any clear correlation between the surface topography (roughness σ_rms_ = 10 nm, crystal thickness 1.3 μm) and the local hotspots. Experimentally, we also establish that the spatial patterns do not change qualitatively at 10 K (Figure S4) suggesting that the hotspots are pinned up to room temperature. As one potential origin of the spatially inhomogeneous Raman signal, we propose that the contact geometry serves as the source of these highly localized patterns. To support this hypothesis, we imaged noncontacted crystals located on the same substrate, i.e., obtained through the very same exfoliation step and subject to the very same fabrication steps. The inset of Figure 4d shows a representative Raman map of such a crystal. The spatial distribution displays a greatly improved homogeneity, and the overall intensity of the additional Raman peak is greatly diminished, albeit still detectable (Figure 4d). We consistently observed the above differences in spatial homogeneity between contacted and noncontacted films across multiple sample batches from different fabrication runs (see Figure S5 for an overview of the measured samples).

In our interpretation, the local hotspots likely arise from spatially inhomogeneous strain patterns in the film. As such, they typically comprise both tensile and compressive biaxial strain components within a typical laser probe volume, explaining the inconclusive observations of the uniaxial strain dependence (Figure 1) and temperature dependence (Figure 2, corresponding to an effective biaxial strain). In the case of contacted films, our experiments suggest that the hotspots’ origin is strain imparted directly in the metallized region. Such strain can originate from the elevated surface temperature of the metal film during and subsequent cooling to the substrate temperature after the evaporation process. For typical physical vapor deposition parameters of contact metals, such as gold,^29,30^ the surface temperature was reported to be anywhere between a few Kelvin^29^ and a few hundred Kelvin^30^ depending on the thermal anchoring of the sample and specific geometry of the evaporator. In principle, the strain is expected to relax away from the contacts in a rather smooth fashion. The experimentally observed, highly localized Raman hot spots at the center of the sample, in between the contacts, points toward a local relaxation and pinning of strain to inhomogeneous profiles. Then, stiffening of the lattice upon cooling or upon application of compressive strain disfavors such local relaxation profiles explaining the suppression of the additional Raman peak. Consistent with this interpretation, we find that laser annealing at room temperature and under ambient conditions with moderate laser powers (*P*_laser_ < 500 μW) reduces the intensity of the additional peak (Figure S6). In this picture, the local laser heating supplies the energy necessary to locally relax the strain patterns. At the same time, this allows us to further exclude surface oxidation as the origin of the spatial inhomogeneities. By contrast, at high laser powers (*P*_laser_ ≥ 1 mW), where visible damage occurs to the films under ambient conditions, we find an irreversible increase of the additional Raman feature. The latter can also be understood as the effect of inhomogeneous strain because AFM measurements reveal significant buckling around the regions damaged by the laser (Figure S7).

## Conclusions

Overall, our results reveal significant variations in the homogeneity of exfoliated HfTe_5_ films, as evidenced by variations in their Raman spectra. Comparing these variations to measurements under external, uniaxial strain, we show that careful Raman microscopy can be used to probe locally inhomogeneous strain profiles, which manifest as additional features in the Raman spectrum of the exfoliated films. As one possible source of these inhomogeneities, we identify, across three independent sample batches (Figure S5) microfabricated electronic contacts, which are a necessity for any further characterization of electronic transport properties. Due to the sensitivity of the transition metal pentatellurides’ electronic structure to lattice strain, our findings have important consequences for electronic transport measurements on such contacted and exfoliated films. Several studies provided evidence, including photoemission spectroscopy and electron transport, that the fundamental gaps of HfTe_5_, and similarly ZrTe_5_, can be strain-tuned across a weak to a strong topological insulator phase.^17,20,21,31^ Moreover, the location of additional, trivial carrier pockets depends on strain as well.^20,31^ Therefore, our results may explain partly the considerable variation of results seen in the literature. While some studies reported a quasi-quantized Hall resistance of gapped Dirac Fermions with well-developed Shubnikov-de-Haas oscillations,^10,15^ other studies reported signatures of anomalous Hall effects with largely suppressed Shubnikov-de-Haas oscillations,^28,32,33^ sometimes even in nominally identical materials, and further studies described the transport features taking into account additional carrier pockets.^34−36^ Based on the substantial inhomogeneity of exfoliated films observed here, it may be advantageous to carefully and systematically differentiate between measurements on bulk crystal (mm-scale) and exfoliated films (μm-scale). We note that, in previous work on HfTe_5_, we already observed such systematic differences in the magnetotransport properties of bulk reference crystals and exfoliated films.^28^ Depending on the used contacts within a multiterminal, exfoliated device, one may probe homogeneous areas with little to no built-in strain or one may probe inhomogeneous areas, which might consist of patches with varying density, varying gap size, or even varying topological nature of the bands. For example, recent studies on ZrTe_5_ found evidence for a nodal-line semimetal phase with a strong magnetochiral anisotropy and nonlinear transport, which were argued to be signatures of spatial inhomogeneity and charge puddling.^37,38^ The latter becomes particularly relevant at the very low carrier densities achievable in these material systems. With these considerations in mind, Raman microscopy at low excitation power (*P*_laser_ ≈ 100 μW) can be a valuable tool to not only track changes in the homogeneity of HfTe_5_ crystals across different fabrication steps but also to select samples or areas within a sample for further advanced characterization based on their homogeneity. While the impact of local disorder potentials and inhomogeneities, arising, for example, from the exfoliation, transfer, fabrication, or growth process, is widely recognized and investigated in strictly two-dimensional van der Waals materials,^39^ it is often implicitly disregarded in three-dimensional, bulk-like van der Waals materials, such as ZrTe_5_, HfTe_5_, Bi_2_Se_3_, or WTe_2_, although substantial inhomogeneities can be present and decisive in three-dimensional systems, as well.^40,41^

## Methods

### Sample Fabrication and Application of Strain

For strain measurements, the HfTe_5_ crystals were mechanically exfoliated onto flexible polyethylene terephthalate (PET) substrates with 125 μm thickness. To avoid sample degradation and ensure a uniform application of strain on the HfTe_5_ crystals, the PET substrate was subsequently spin-coated with 400 nm of poly(methyl methacrylate) (PMMA). The applied strain ϵ is estimated according to ref (42) using ϵ = τ sin θ /2*a*, where τ is the thickness of the flexible substrate, θ is the angle of the tangent at the minimum strain point, and 2*a* is the separation of the bent substrate edges. For samples with electronic contacts, as used in typical transport measurements, we utilized a maskless lithography tool to pattern the contact geometries. To remove the natural surface oxide that forms during exposure of the HfTe_5_ crystals to ambient conditions, Ar-sputtering was performed prior to the deposition of 25 nm titanium (Ti) and 350 nm gold (Au).

### Raman Spectroscopy

The Raman measurements were carried out using a commercial Raman microscope (Alpha300R). A 532 nm laser was focused parallel to the *b*-axis of the fabricated devices. The laser was polarized linearly along the *a*-axis, and the scattered light was collected with an unpolarized detection. The spectra in Figure 1 were acquired under ambient conditions using a 50× long-working distance objective (NA = 0.55). The spectra in Figures 2 and 3 were acquired in a flow cryostat using a 63× objective (NA = 0.75, cover glass correction). The spectra in Figure 4 were acquired under ambient conditions using a 100× objective (NA = 0.9). To minimize the impact of local laser heating and to prevent degradation of the crystals by oxidation, all Raman spectra were taken at 100 μW. Unless stated otherwise, the spectra were taken using an 1800 lines/mm grating.

## Supporting Information Available

Impact of laser-induced heating on Raman modes, temperature dependence of Raman intensities, statistical correlations between Raman and AFM, Raman map at 10 K, overview of samples, impact of prolonged laser exposure on topography, and Raman spectrum (PDF)
