# Supplementary material for: Probing the Spatial Homogeneity of Exfoliated HfTe5 Films
Source: ACS Nano. 2024 Jul 3;18(28):18327–33. doi: 10.1021/acsnano.4c02081 (PMC11256895; doi:10.1021/acsnano.4c02081)
Supplement: Supplementary file 1 — nn4c02081_si_001.pdf [file nn4c02081_si_001.pdf]

Supporting Information for
Probing the spatial homogeneity of exfoliated $HfTe_5$ films

Maanwinder P. Singh,[1,2] Qingxin Dong,[3,4] Gen-Fu Chen,[3,4,5] Alexander W. Holleitner,[1,2] and Christoph Kastl[1,2]

[1] *Walter Schottky Institut, Technical University of Munich, Am Coulombwall 4a, 85748 Garching, Germany.*
[2] *Munich Center for Quantum Science and Technology (MCQST), Schellingstr. 4, 80799 Munich, Germany.*
[3] *Institute of Physics and Beijing National Laboratory for Condensed Matter Physics, Chinese Academy of Sciences, 100190 Beijing, China.*
[4] *School of Physical Sciences, University of Chinese Academy of Sciences, 100049 Beijing, China.*
[5] *Songshan Lake Materials Laboratory, Dongguan, 523808 Guangdong, China.*

CONTENTS

S1: Laser power-induced heating and corresponding temperature-induced shift of Raman Modes 2

S2: Temperature dependence of Raman intensities 2

S3: Visualization of statistical correlations between Raman intensity and AFM topography 3

S4: Raman map of an exfoliated $HfTe_5$ crystal at 10 K 3

S5: Overview of three different sample batches evaluated in our study 4

S6: Systematic time-dependent study of the Raman spectrum at different laser powers 5

S7: AFM topography map of an uncontacted $HfTe_5$ crystal with local laser damage 6

S1: LASER POWER-INDUCED HEATING AND CORRESPONDING TEMPERATURE-INDUCED SHIFT OF RAMAN MODES

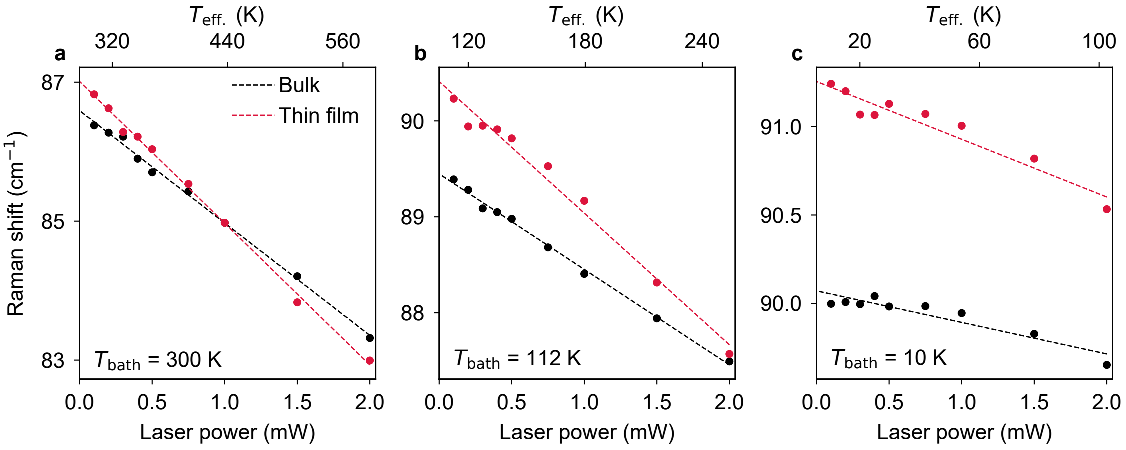

FIG. S1. **Laser power-induced heating and corresponding temperature-induced shift of Raman Modes.** Variation of the $B_{2g}^{(2)}$ mode with laser power at a substrate temperature of **a** 300 K, **b** 112 K, and **c** 10 K for the bulk reference (black dots) and an exfoliated film. The plots include a secondary x-axis on the top which represents the effective sample temperature within the laser spot T_{eff}. This effective temperature was estimated by comparing the peak position as function of laser power to the peak position as function of substrate temperature. The latter was measured at a low power of 100 µW. The true value of the Raman peak position at a given substrate temperature is then estimated by extrapolating the power dependence to zero laser power.

S2: TEMPERATURE DEPENDENCE OF RAMAN INTENSITIES

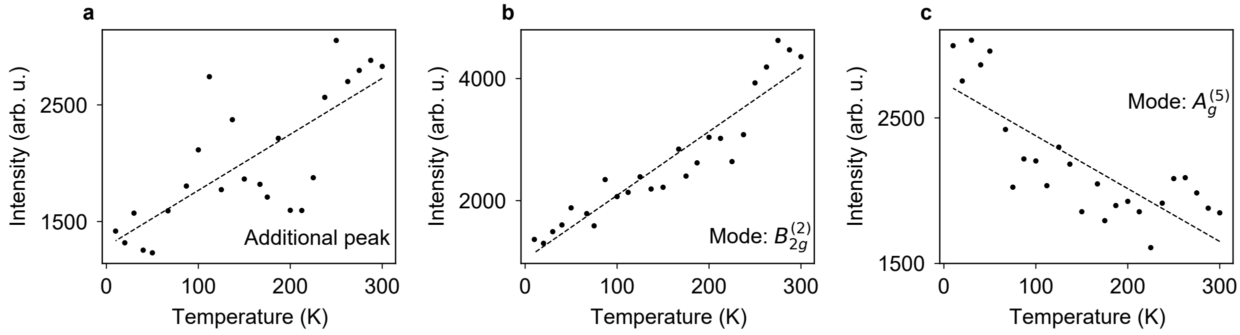

FIG. S2. **Temperature dependence of Raman intensities.** **a** The overall intensity of the additional, strain-sensitive peak increases with temperature. **b** Similarly, the intensity of the $B_{2g}^{(2)}$ mode increases with temperature. **c** By contrast, the intensity of the $A_g^{(5)}$ mode decreases with temperature. We define the intensity as the area under the peak. The intensities were calculated by numerically integrating the spectra from 118 cm^{-1} to 138 cm^{-1}, 78 cm^{-1} to 98 cm^{-1} and 165 cm^{-1} to 175 cm^{-1}, respectively.

S3: VISUALIZATION OF STATISTICAL CORRELATIONS BETWEEN RAMAN INTENSITY AND AFM TOPOGRAPHY

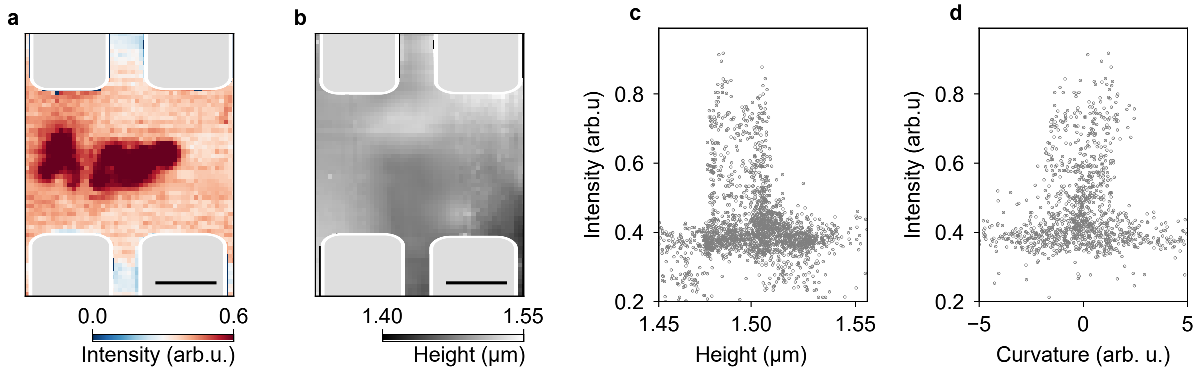

FIG. S3. **Visualization of statistical correlations between Raman intensity and AFM topography.** **a** Spatial map of Raman intensity integrated from 118 cm^{-1} to 138 cm^{-1}. **b** Spatial map of the sample topography obtained via AFM. Scale bars are 5 µm. **c** Scatter plot correlating the integrated Raman intensity and AFM topography at each respective pixel in **a** and **b**. To allow for precise comparison, the AFM data was down-sampled and aligned to match the position and resolution of the Raman measurements. Raman intensity and topography do not show any clear correlation. **d** Correlation plot of integrated Raman intensity and local topography curvature. The local curvature was approximated using a second-order Gaussian derivative filter. The finite slope of the clustered data points indicates a possible correlation, consistent with the impact of local strain profiles.

S4: RAMAN MAP OF AN EXFOLIATED HFTE$_5$ CRYSTAL AT 10 K

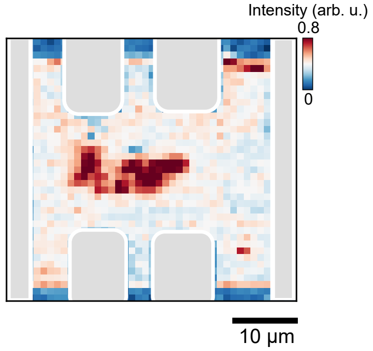

FIG. S4. **Raman map of an exfoliated HfTe$_5$ crystal at 10 K.** Raman intensity map of the contacted sample at 10 K. The intensity was integrated within the spectral range from 118 cm^{-1} to 138 cm^{-1}. The grey shaded areas denote the gold contacts. The sample is the same as shown in Figure 4a of the main manuscript.

S5: OVERVIEW OF THREE DIFFERENT SAMPLE BATCHES EVALUATED IN OUR STUDY

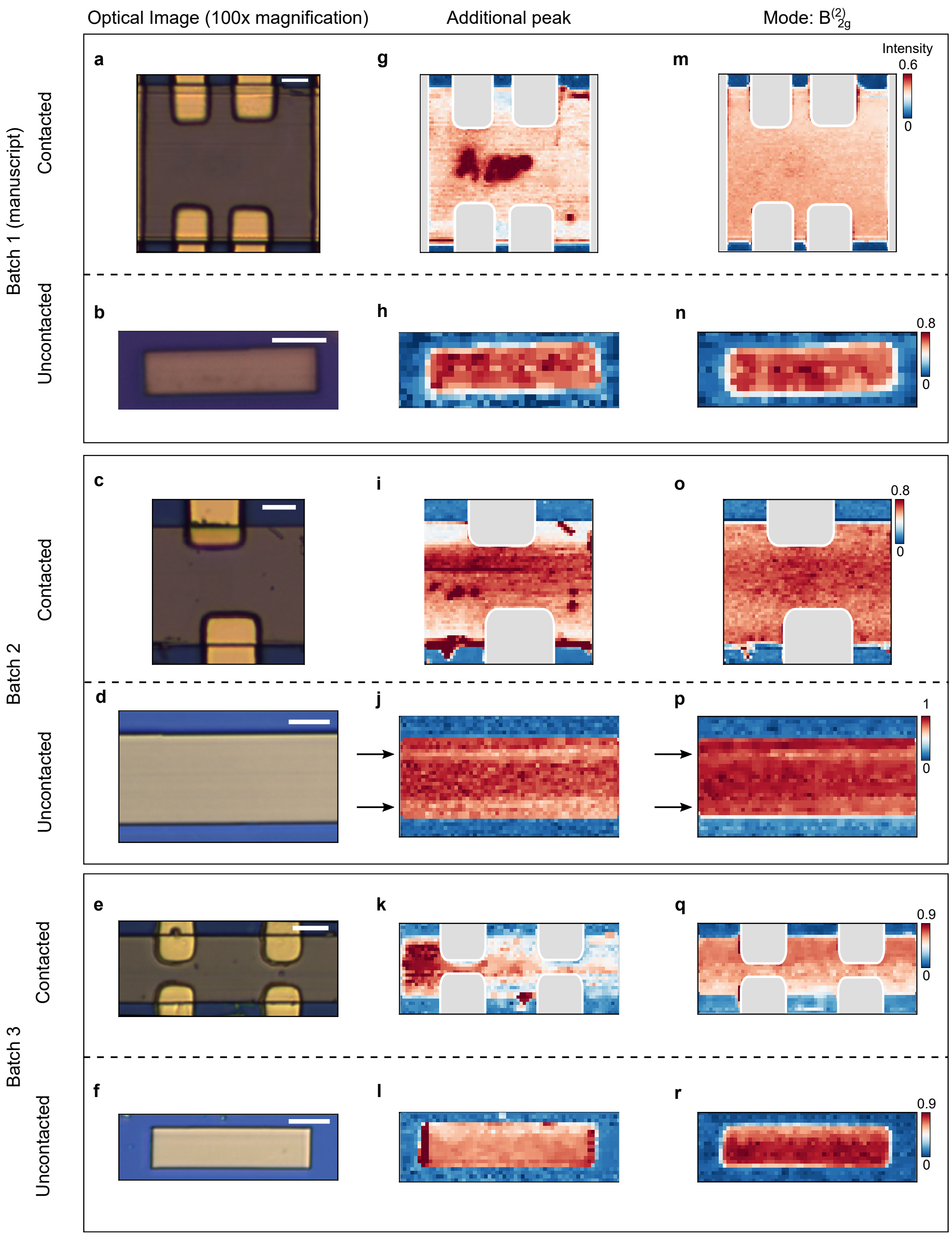

FIG. S5. **Overview of three different sample batches evaluated in our study.** For each batch, we compared a contacted film and a non-contacted film located on the same substrate. In this way, we ensured that the contacted and non-contacted films experienced identical nanofabrication processing. For each batch, we show **a-f** optical images, **g-l** Raman maps integrated from 118 cm^{-1} to 138 cm^{-1} (additional, strain-sensitive peak), and **m-r** Raman maps integrated from 60 cm^{-1} to 110 cm^{-1} ($B^{(2)}_{2g}$ mode). The Raman maps corresponding to the $B^{(2)}_{2g}$ mode appear homogeneous in the case of both contacted and uncontacted samples. For the additional strain-sensitive peak, substantial inhomogeneity appears exclusively in the case of contacted samples. Some Raman measurements show horizontal, correlated line artifacts, e.g., arrows in **j** and **p**. These are caused by small focus drifts during the measurement time (several hours). The thicknesses of the contacted samples are **a** 1.3 µm, **c** 600 nm, and **e** 470 nm as determined by AFM. All scale bars are 5 µm. Batch 1 (**a, b, g, h, m,n**) corresponds to the data shown in the main manuscript (Figure 2, Figure 3 and Figure 4).

S6: SYSTEMATIC TIME-DEPENDENT STUDY OF THE RAMAN SPECTRUM AT DIFFERENT LASER POWERS

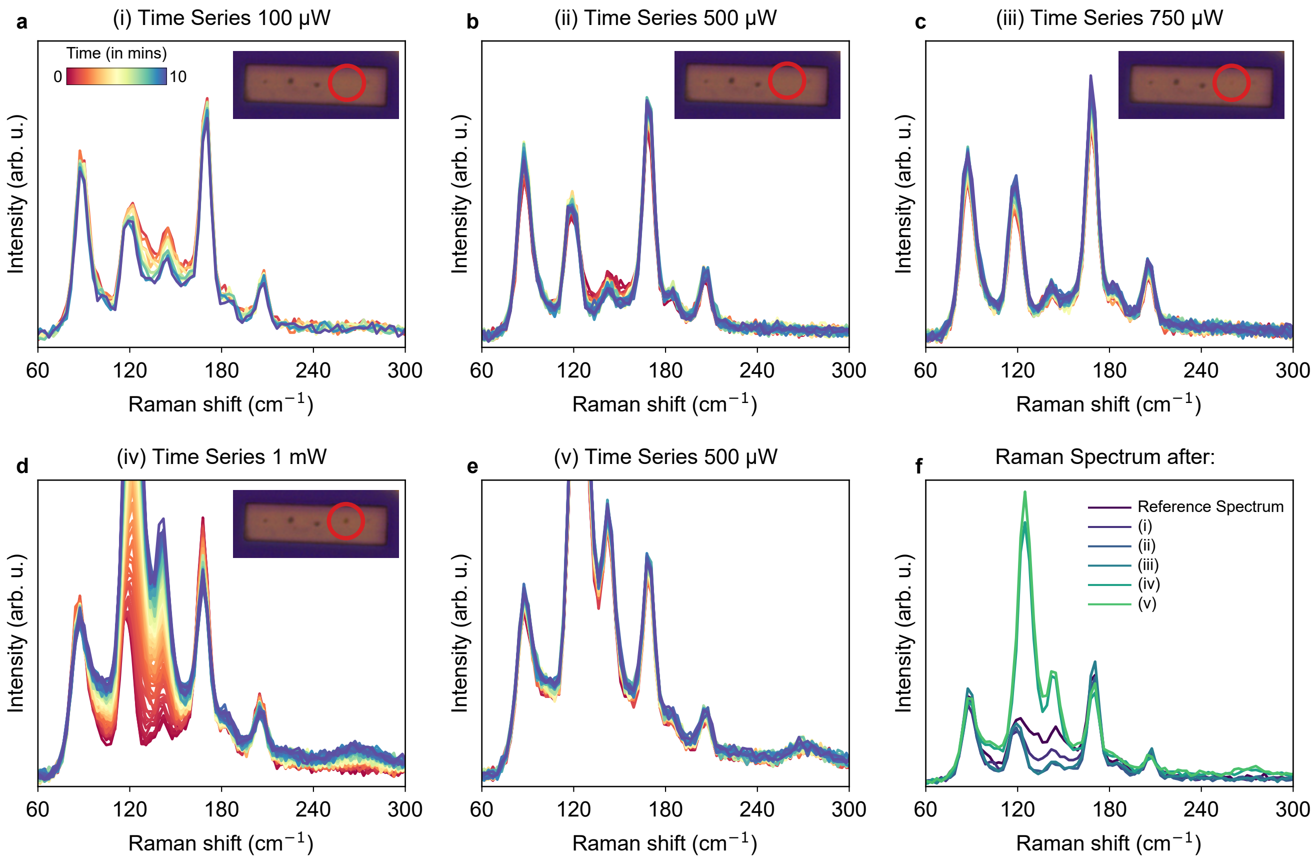

FIG. S6. **Systematic time-dependent study of the Raman spectrum at different laser powers.** A series of time-dependent measurements, each spanning 10 minutes, conducted at distinct laser powers, **a** $P_{\mathrm{laser}} = 100\,\mu\mathrm{W}$, **b** $500\,\mu\mathrm{W}$, **c** $750\,\mu\mathrm{W}$ and **d** $1\,\mathrm{mW}$. All spectra where taken at the location marked by the red circle in the inset. For laser powers up to $500\,\mu\mathrm{W}$ (i and ii), the intensity of the additional peak decreases with prolonged illumination time, and no laser-induced damage is visible in the optical images. This regime can interpreted as local laser annealing resulting in local strain relaxation. Starting at $750\,\mu\mathrm{W}$, the intensity of the additional peak begins to rise again and laser damage becomes apparent in the optical images as a light "burn spot". Beyond $1\,\mathrm{mW}$, the intensity of the additional peak undergoes a significant, irreversible increase and a dark "burn spot" becomes visible. Additionally, another Raman peak appears around 270 cm^{-1}, suggesting substantial oxidation/degradation in the exfoliated film. **e** Continuing the time series at a reduced laser power ($P_{\mathrm{laser}} = 500\,\mu\mathrm{W}$) no longer reduces the intensity of the additional Raman peaks showing the irreversibility of the degradation process at laser powers beyond $1\,\mathrm{mW}$. **f** Comparison of the reference Raman signal, which was obtained prior to the time series, with Raman spectra recorded after each time series. Each spectrum was measured at $P_{\mathrm{laser}} = 100\,\mu\mathrm{W}$ for better comparison. All data were measured in a spectrometer with a 600 lines/mm grating.

S7: AFM TOPOGRAPHY MAP OF AN UNCONTACTED $HfTe_5$ CRYSTAL WITH LOCAL LASER DAMAGE

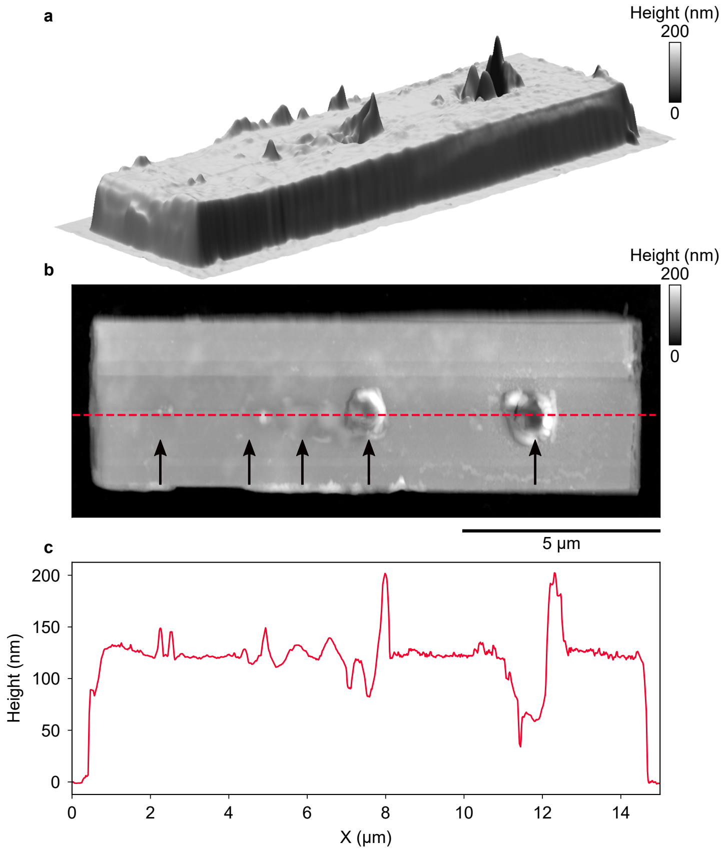

FIG. S7. **AFM topography map of an uncontacted $HfTe_5$ crystal with local laser damage.** **a** Three-dimensional visualization of the topography. Points on the sample that were damaged by the laser are surrounded by an elevated halo. **b** Two-dimensional visualization of the same topography map. **c** Line profile along the dashed line in **b**. The crystal thickness has been measured to be approximately 120 nm. The damaged spots (as indicated by the arrows) comprise both depressed and elevated regions, suggesting a locally inhomogeneous strain profile with both tensile and compressive components.
